# The Rhetoric of Decolonizing Global Health Fails to Address the Reality of Settler Colonialism: Gaza as a Case in Point

**DOI:** 10.34172/ijhpm.2024.8419

**Published:** 2024-04-09

**Authors:** Eivind Engebretsen, Mona Baker

**Affiliations:** Sustainable Health Unit, University of Oslo, Oslo, Norway.

**Keywords:** Global Health, Decolonization, Settler Colonialism, Gaza, Palestine, Politics of Health

## Abstract

This editorial critiques the existing literature on decolonizing global health, using the current assault on health in Gaza as a case in point. It argues that the failure to address the ongoing violence and blatant targeting of health facilities, personnel and innocent civilians demonstrates most clearly the limitations of an approach that is strong on rhetoric and weak on mounting a forthright challenge to the entire system supporting and perpetuating settler colonialism. We propose a more radical rethinking of the position of global health institutions within the current neoliberal system and of the systems of knowledge production that continue to underpin the existing colonial approach to the health of victims of settler colonialism.

 Originally conceptualized as a commitment to improving health and achieving health equity across the globe, the term ‘global health’ has experienced significant shifts in its application. As observed by Smeeth and Kyobutungi, global health has often been portrayed in practice as an apolitical strategy, primarily involving “people or institutions from high-income countries taking an interest in the health of people in low-income and middle-income countries (LMICs).”^1^ This approach, devoid of political considerations, has faced considerable criticism recently, especially within the context of diverse efforts aimed at “decolonizing global health.” Some of this critical literature has engaged with the idea of decolonization by drawing on decolonial theory and social theories developed outside the field of global health; scholars working in this area have called for a radical transformation of the field’s epistemological and material framework.^2,3^ Conversely, many interpretations of decolonization focus more on reforming the field or specific aspects of it, such as educational curricula and development policies.^4,5^ Here, “decolonizing” is often used “interchangeably with the liberal notion of ‘diversifying,’ emptying the historical context and conceptual meanings of decolonization as rooted in the material struggles for liberation against European imperialism.”^6^ While the former approach is transformational, calling for an overhaul of existing structures, the latter is reformist, advocating for changes within the existing neoliberal, capitalist framework.

 The co-optation of decolonization initiatives has significantly diluted the potency of the agenda of decolonizing global health. Too often, these initiatives are restricted to symbolic actions which serve to mask the ongoing material manifestations of colonialism in the global system. In the same vein, coloniality is simplified to a mere passive extension of historical colonial rule, rather than being recognized as a network of globally enmeshed relations that continue to support racial capitalism, colonialism, and imperialism across various global landscapes.^6^ At best, such initiatives have become instruments for advocating modest change; at worst, they have acted as facades for perpetuating oppression by promoting a “settler move to innocence”^7^: a discourse that allows its advocates to disassociate from a settler identity while continuing to enjoy settler privileges and inhabit colonized land. As the current situation in Gaza demonstrates most powerfully, calls for decolonizing global health have ultimately proved no more than window dressing. The decolonization approach has failed to address the enduring violence and oppression perpetuated by the global political economy, which is underpinned by colonial and capitalist ideologies.

 Israel’s systematic destruction of healthcare infrastructure and killing of many health personnel in Gaza since 7 October,^8^ without the World Health Organization (WHO) and other institutions located in the Global North being able to offer much more than increasingly desperate calls for a ceasefire and aid to be allowed through, throws the failure of this decolonizing approach into sharp relief. The apparent powerlessness of these institutions in the face of overwhelming violence and obstruction by powerful governments highlights the necessity for global health to address the nature of settler colonialism in the 21st century and its own imbrication within the structures that sustain these colonial practices. In particular, we argue, a serious call for decolonization in global health requires a more radical approach that transcends mere institutional reform, one in which “decolonial thought” is “connected to anti-colonial, anti-capitalist, and anti-imperial *action.*”^9^ This requires us to align our discourse and action with those of anti-hierarchical movements, particularly in the region where settler colonial practices are at their most abhorrent—namely, Palestine. Addressing this issue also necessitates a deep reevaluation of the current politics of knowledge in the field of global health. This field is marked by a traditional top-down “knowledge translation” approach, largely dominated by Global North institutions that serve as gatekeepers of knowledge, leading to the marginalization and suppression of local insights, notably from regions such as Palestine.

## The Limitations of the Decolonizing Approach to Global Health

 The current assault on Gaza offers a stark illustration of the systemic straightjacket within which global health organizations such as WHO operate and the continued failure of the decolonization movement to address the real issue of their entanglement with settler colonial logics and processes. Health facilities and personnel were systematically targeted well before the events of 7 October. In May 2021 alone, Israeli airstrikes damaged six hospitals and nine primary healthcare centres, the COVID-19 laboratory and the Ministry of Health were both hit, and at least two doctors were killed.^10^ Since 7 October, at least 22 hospitals, 51 primary healthcare centres and 59 ambulances were destroyed, and a staggering 565 medical workers, including 106 doctors, were killed.^11^ By 20 December, not a single hospital in Gaza was fully functional.^12^ This is not to mention the number of children killed, or the 9000 children who have had a limb amputated, with many of these having to endure amputation without anesthesia, according to the United Nations International Children’s Emergency Fund (UNICEF).^13^

 Despite recognizing the political context of the assault on Gaza and the West Bank, the WHO’s approach to health in the occupied Palestinian territories systematically overlooks the crucial historical connection to settler colonialism. Unlike its commentary on the violence in Ukraine, practically all WHO’s repeated condemnations of the attacks on health in Gaza are formulated in the passive tense, rarely naming the aggressor explicitly. For decades, WHO refrained from questioning the colonial structures that perpetuate such intensive assaults on the health of an entire population, choosing instead to treat the aftermath of colonial violence as a humanitarian crisis. This approach has now forced the organization to confront the fact that “it will be all but impossible to improve the ‘catastrophic’ health situation in Gaza” in future.^14^ The Chair of Global and Public Health at the University of Edinburgh has warned of “almost a quarter of Gaza’s 2 million population—close to half a million human beings—dying within a year.”^15^ How and why did our global health organizations allow the situation to deteriorate to this extent? What role has the empty rhetoric of decolonization played in diverting our attention from the massive, radical change in outlook and practice required for global health to address the ugly realities of settler colonialism? This is as much a political as it is a medical question, if the two can be meaningfully separated.

 The rhetoric of decolonizing global health, then, has done nothing to address the root causes of the disastrous health situation in Gaza and the West Bank. In addition to examining the financial, operational, and political dependence of global health institutions on governments that have a long history of involvement in settler colonialism, addressing this issue, we argue, also necessitates a profound reassessment of the prevailing politics of knowledge within the field. As Qato has pointed out, “we must challenge the logics of research itself, the epidemiological models upon which this research is built, and the data upon which policies are enacted and imagined.”^16^ The domain of health is currently characterized by a conventional top-down method of “knowledge translation,” where supposedly universal interventions are modified for use in the cultures of the Global South.^17^ This process of adapting knowledge produced in the Global North and presumed universal to the needs of the Global South is frequently blind to the underlying power disparities between North and South, and particularly blind to the context of settler colonialism. Just as the mere provision of medical care does not ensure justice, neither does the presence of biomedical evidence equate to justice.^16^ Indeed, evidence can be manipulated to mask and rationalize ongoing disparities, allowing the inherent and ever-present colonial violence to persist unchallenged. This is further exacerbated by the location of major outlets for debate on global health issues within a neoliberal, capitalist infrastructure that is particularly susceptible to pressure from those protecting settler colonial interests. Examples abound of research, opinion pieces and letters on Palestine and by Palestinians being removed from websites of *The Lancet, BMJ *and similar venues following pressure from “readers.”^18,19^ Such widespread practices of censorship serve to ensure the erasure of Palestinian voices and experiences from global health research, including research on the health situation in Palestine. Beyond direct censorship, the root “cause of causes” of ill health in Palestine, namely settler colonial practices, is carefully camouflaged in continuous acts of discursive complicity that treat connected health determinants as separate phenomena, to be studied under categories such as “refugee health,” “minority health” and “conflict health,” thus ensuring that Israel is not explicitly identified as accountable and responsible for Palestinian ill health.^20^ A truly decolonizing approach to knowledge translation would protect and prioritize the experience of the subjects of settler colonial violence as the foundational element for advancements in global health, and would avoid the fragmentation of causes of their ill health into categories that mask the responsibility of their occupiers.

## Towards a Decolonizing Approach to Knowledge Translation

 Commenting on settler colonialism and health in Palestine, Selcer and Surya rightly observe that “healthcare institutions do not operate in an abstract world” but are deeply entrenched in the political realities of conflict, inequality and injustice.^21^ A decolonizing approach to knowledge translation must therefore extend beyond reassessing abstract epistemological frameworks; it must be prepared to confront and dismantle the colonial infrastructures that support the health knowledge system. Moreover, decolonial discourses must move beyond the oversimplified linear model that progresses from knowledge to action, where global health is initially decolonized theoretically before being applied to new social struggles. Instead, that linear model must be replaced by a dynamic interplay of action, reflection and theorization that is deeply rooted within the fabric of social struggle. Rather than remaining within its safe, risk-free theoretical bounds, decolonial work should be clearly defined and realized through direct engagement with the victims of settler colonialism, and on *their* terms.

 The decolonization framework challenges the notion of the knowledge source as a universally applicable gold standard, particularly when implemented in contexts in the Global South, by arguing that this concept requires dismantling. Yet, we contend that this method paradoxically relies on the very knowledge-action structure it aims to critique. It presupposes that knowledge can be decolonized in theory and in isolation – through a self-decolonizing process initiated and conducted by the Global North. This editorial advocates for confronting this method with an action-to-knowledge strategy (referred to elsewhere as a translational medical humanities approach).^22^ This alternative strategy is poised to actively confront and dismantle the *material* colonial infrastructures underpinning the health knowledge system, while fundamentally grounding knowledge creation in the grassroots realities of social conflict and human suffering.

## Ethical issues

 Not applicable.

## Competing interests

 Authors declare that they have no competing interests.
